# Draft Genome Sequence of the Alga-Aggregating Bacterium *Bacillus* sp. Strain RP1137

**DOI:** 10.1128/genomeA.00973-13

**Published:** 2014-01-02

**Authors:** Ryan J. Powell, Tsvetan R. Bachvaroff, Russell T. Hill

**Affiliations:** Institute of Marine and Environmental Technology, University of Maryland Center for Environmental Science, Baltimore, Maryland, USA

## Abstract

*Bacillus* sp. strain RP1137 is a bacterium that is able to rapidly and efficiently aggregate biofuel-producing microalgae. By 16S rRNA gene sequencing, it was found to be related to the industrially important *Bacillus megaterium*. Here, we report the draft genome sequence of *Bacillus* sp. strain RP1137.

## GENOME ANNOUNCEMENT

The development of algal biofuels is a promising means for sustainably replacing fossil fuels. One of the economic challenges of producing algal biofuel is being able to efficiently harvesting microalgae from the water in which they are growing. *Bacillus* sp. strain RP1137 is a bacterium that is able to rapidly and efficiently aggregate several biofuel-producing microalgae (1). The bacterium was discovered as a contaminant in the culture of another alga-aggregating bacterium. RP1137 is most closely related to *Bacillus megaterium* strain PPB7 by 16S rRNA gene sequencing (1).

Bacteria within the *B. megaterium* clade are industrially important organisms. They have been used for the production of penicillin amidase and steroid hydrolases and the aerobic production of vitamin B_12_ (2). Organisms within this group are often genetically tractable and amenable to large-scale fermentation (2). *B. megaterium* species are cosmopolitan and can be isolated from sediment, seawater, and freshwater and are found to be associated with eukaryotes (2).

The genomic DNA from RP1137 was extracted from an overnight culture grown in marine broth 2216 (BD, Franklin Lakes, NJ) at 30°C in a 125-ml Erlenmeyer flask with shaking at 180 rpm. DNA was extracted using an UltraClean microbial DNA isolation kit (MO-BIO, Carlsbad, CA.). The DNA was sequenced using the Nextera XT kit with 250-bp paired-end read sequencing on an Illumina MiSeq. The 7,878,212 reads comprise a total of 1,831,418,429 nucleotides. The reads were assembled and processed using CLC Genomics Workbench and the PSU galaxy server (3–5), and resulted in 60 contigs of >1,000 bp with 435-fold average coverage. The total size of the resulting assembly was 5,878,785 bases, with a G+C content of 37.5%. The annotation and prediction of genes were done using the Rapid Annotations using Subsystems Technology (RAST) server (6). A total of 6,159 coding sequences were predicted and 14 rRNAs and 92 tRNAs were annotated.

The sequence data will be used to help elucidate the aggregation mechanism of RP1137 and to guide directed genetic modification of the strain to increase the efficiency of the aggregation phenotype.

### Nucleotide sequence accession number.

The draft genome sequence of *Bacillus* sp. strain RP1137 was deposited in DDBJ/EMBL/GenBank under the accession no. AXZS00000000.
